# Supplementary material for: Mitofilin–mtDNA Axis Mediates Chronic Lead Exposure-Induced Synaptic Plasticity Impairment of Hippocampal and Cognitive Deficits
Source: Biomolecules. 2025 Feb 12;15(2):272. doi: 10.3390/biom15020272 (PMC11852649; doi:10.3390/biom15020272)
Supplement: Supplementary file 1 [file biomolecules-15-00272-s001.zip › biomolecules-3357300-supplementary.pdf]

WB image

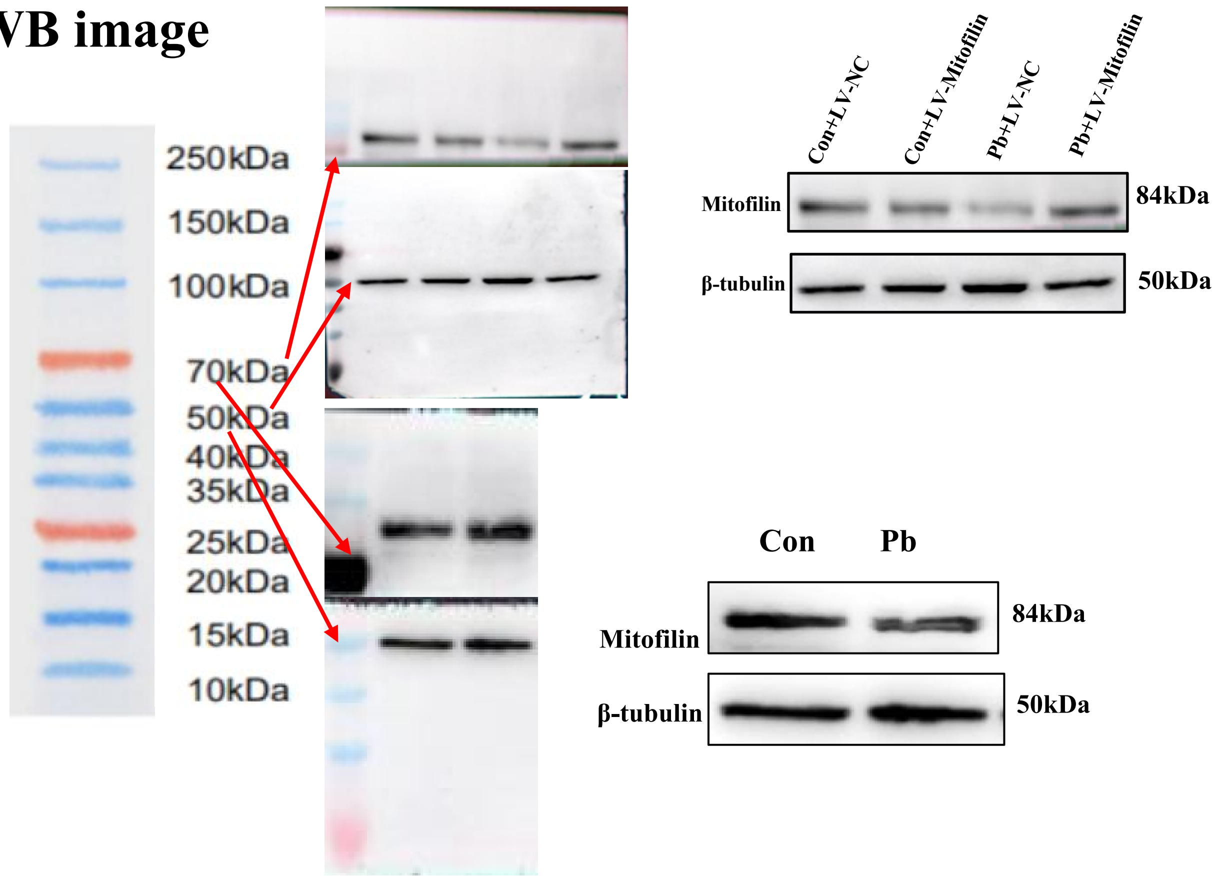
250kDa
150kDa
100kDa
70kDa
50kDa
40kDa
35kDa
25kDa
20kDa
15kDa
10kDa
Con+LV-NC
Con+LV-Mitofilin
Pb+LV-NC
Pb+LV-Mitofilin
Mitofilin
84kDa
β-tubulin
50kDa
Con
Pb
Mitofilin
84kDa
β-tubulin
50kDa

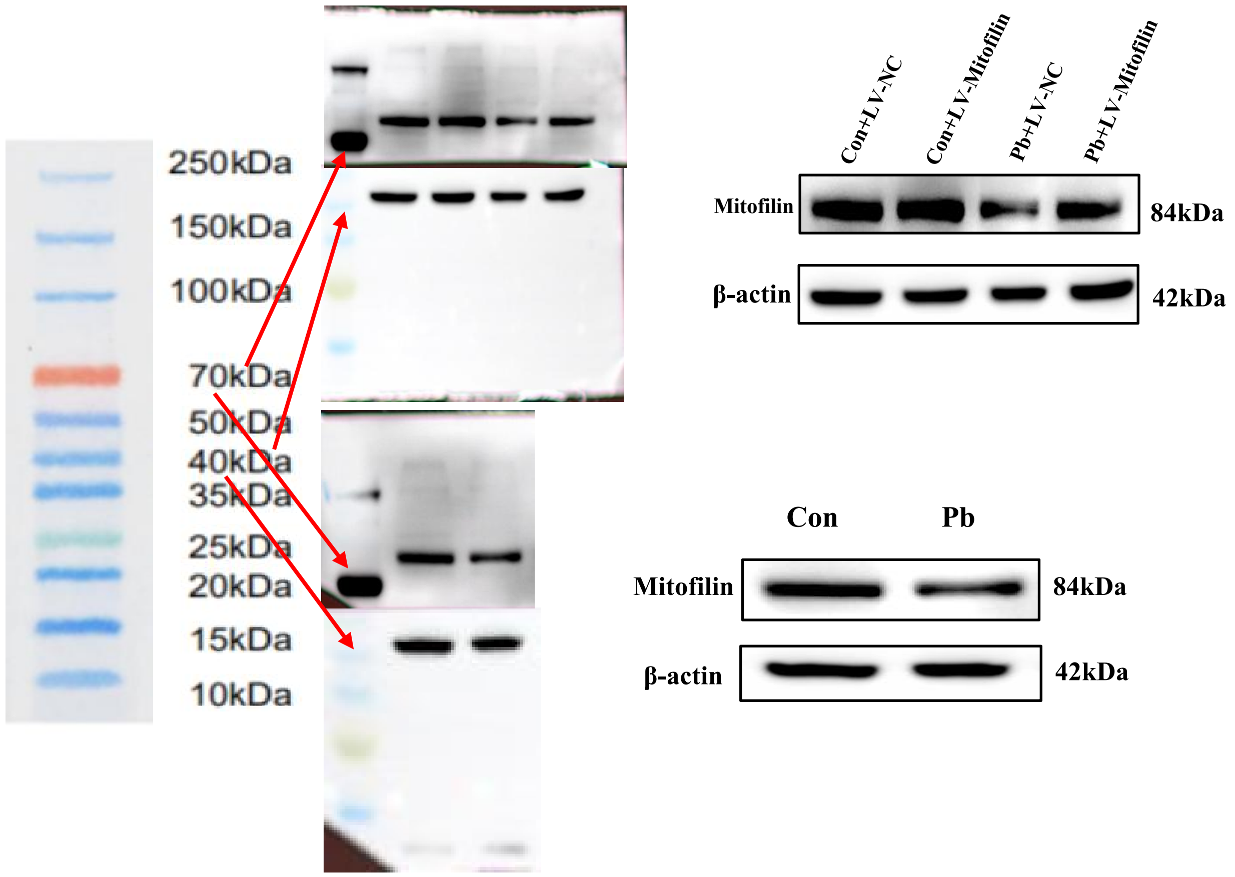
250kDa
150kDa
100kDa
70kDa
50kDa
40kDa
35kDa
25kDa
20kDa
15kDa
10kDa
Con+LV-NC
Con+LV-Mitofilin
Pb+LV-NC
Pb+LV-Mitofilin
Mitofilin
84kDa
β-actin
42kDa
Con
Pb
Mitofilin
84kDa
β-actin
42kDa

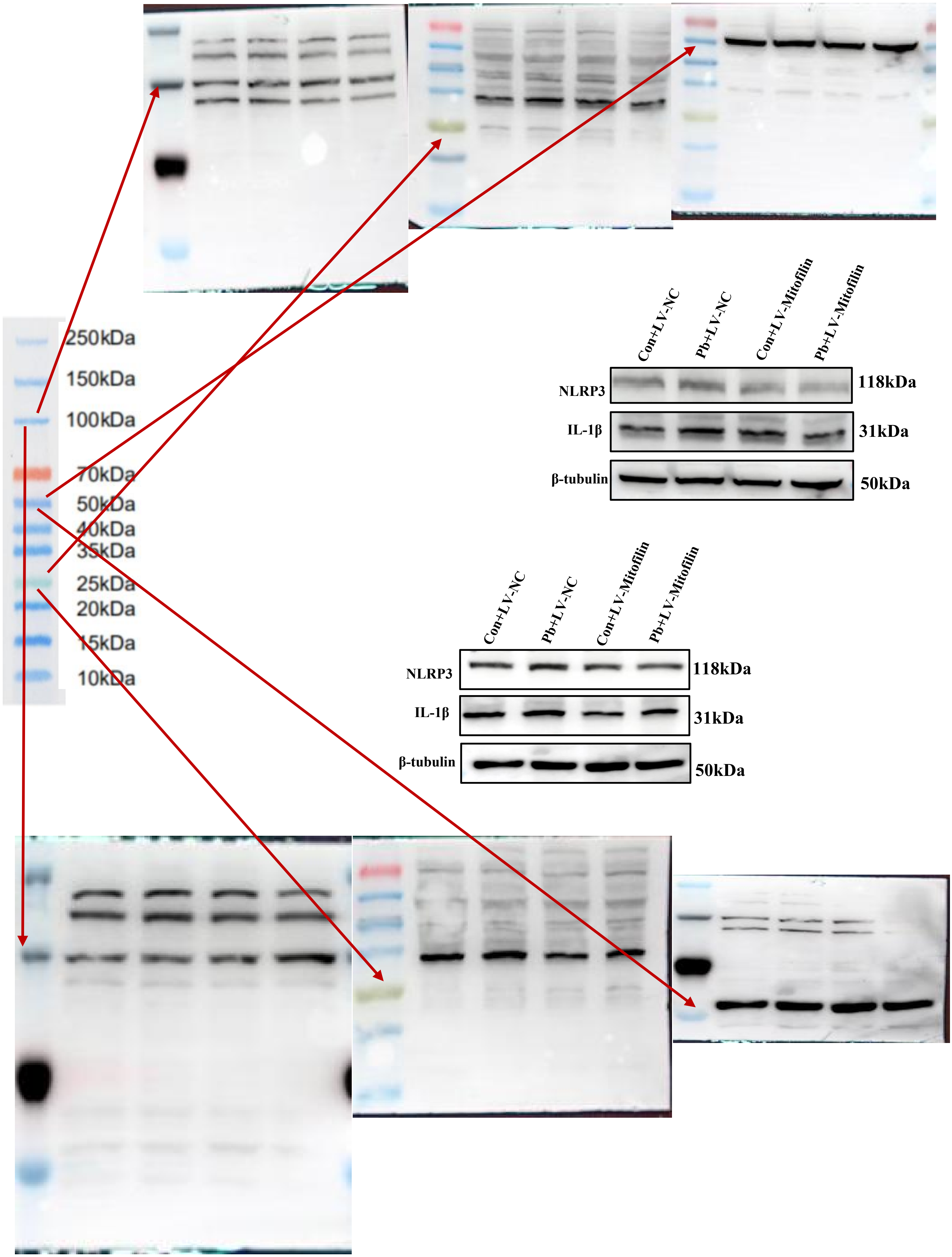

250kDa
150kDa
100kDa
70kDa
50kDa
40kDa
35kDa
25kDa
20kDa
15kDa
10kDa
Con+LV-NC
Pb+LV-NC
Con+LV-Mitofilin
Pb+LV-Mitofilin
NLRP3
118kDa
IL-1β
31kDa
β-tubulin
50kDa
Con+LV-NC
Pb+LV-NC
Con+LV-Mitofilin
Pb+LV-Mitofilin
NLRP3
118kDa
IL-1β
31kDa
β-tubulin
50kDa

Transmission electron microscope image

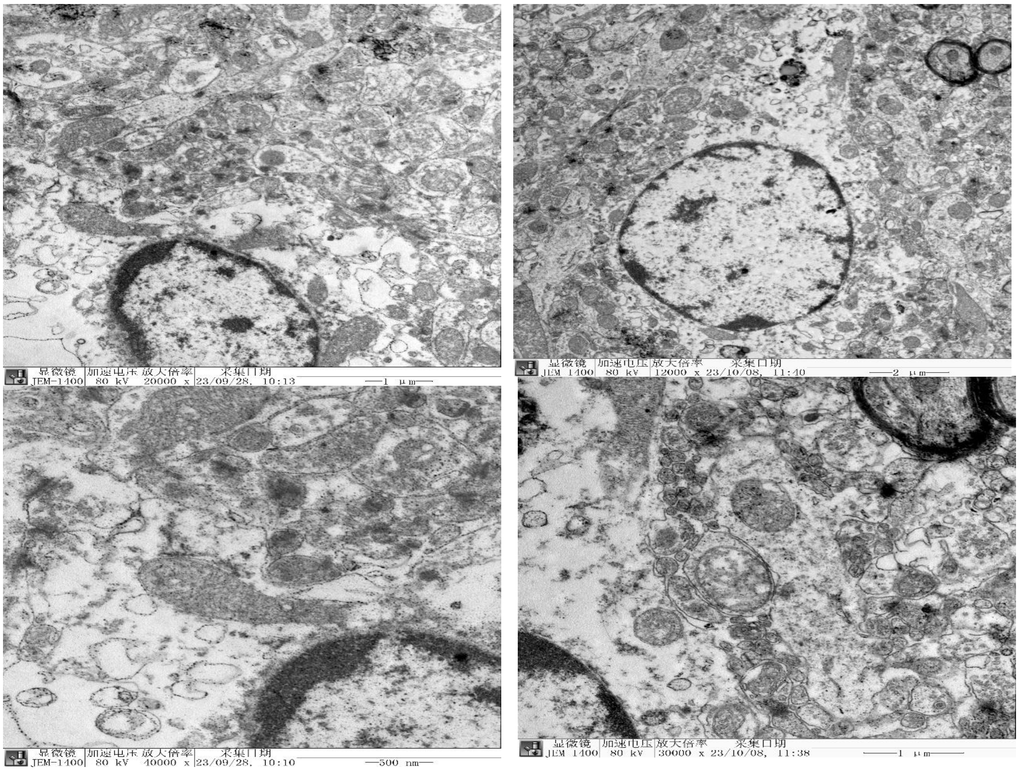

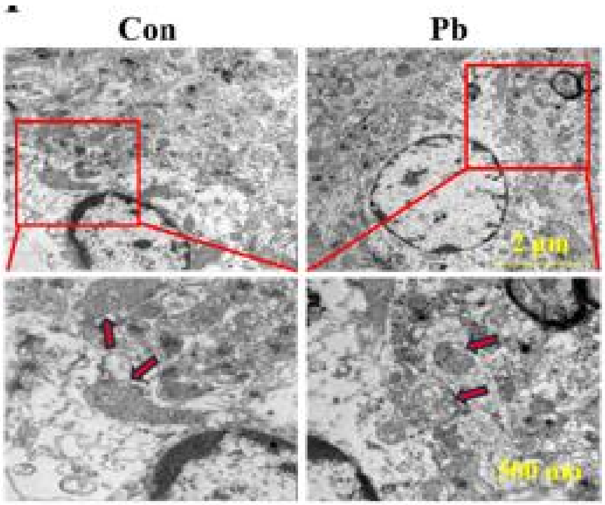

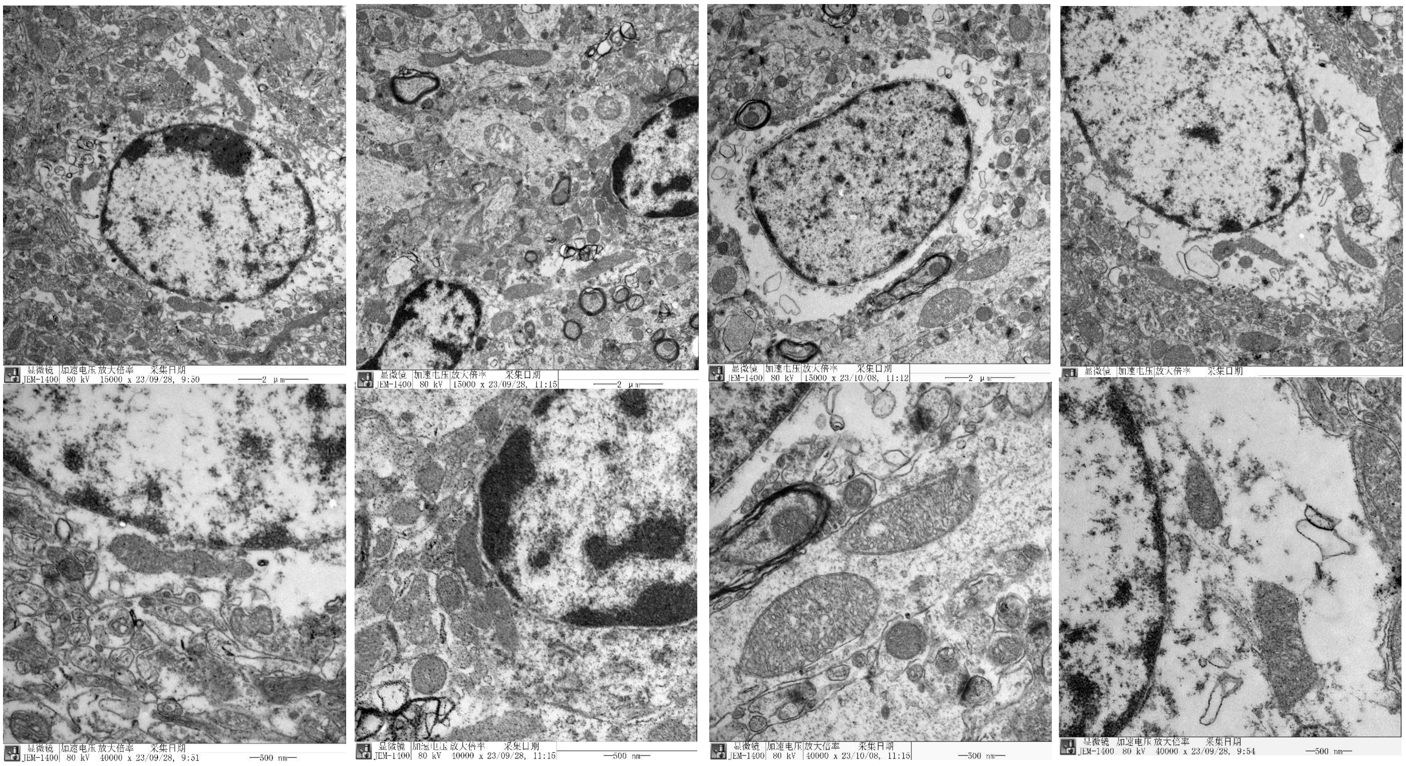

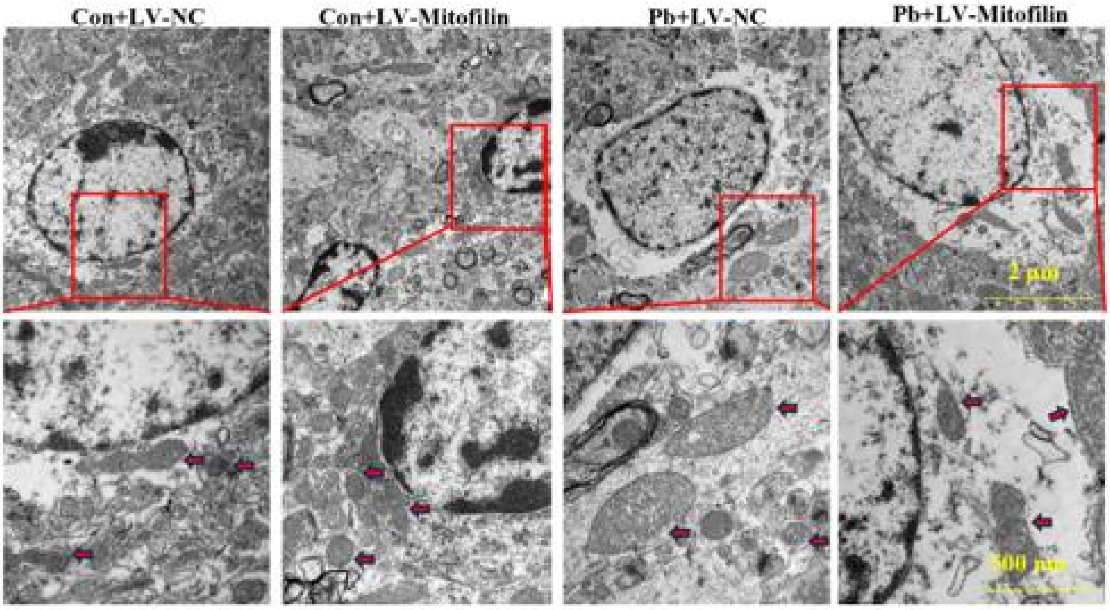

Con+LV-NC
Con+LV-Mitofilin
Pb+LV-NC
Pb+LV-Mitofilin
2 μm
500 nm

Immunofluorescence

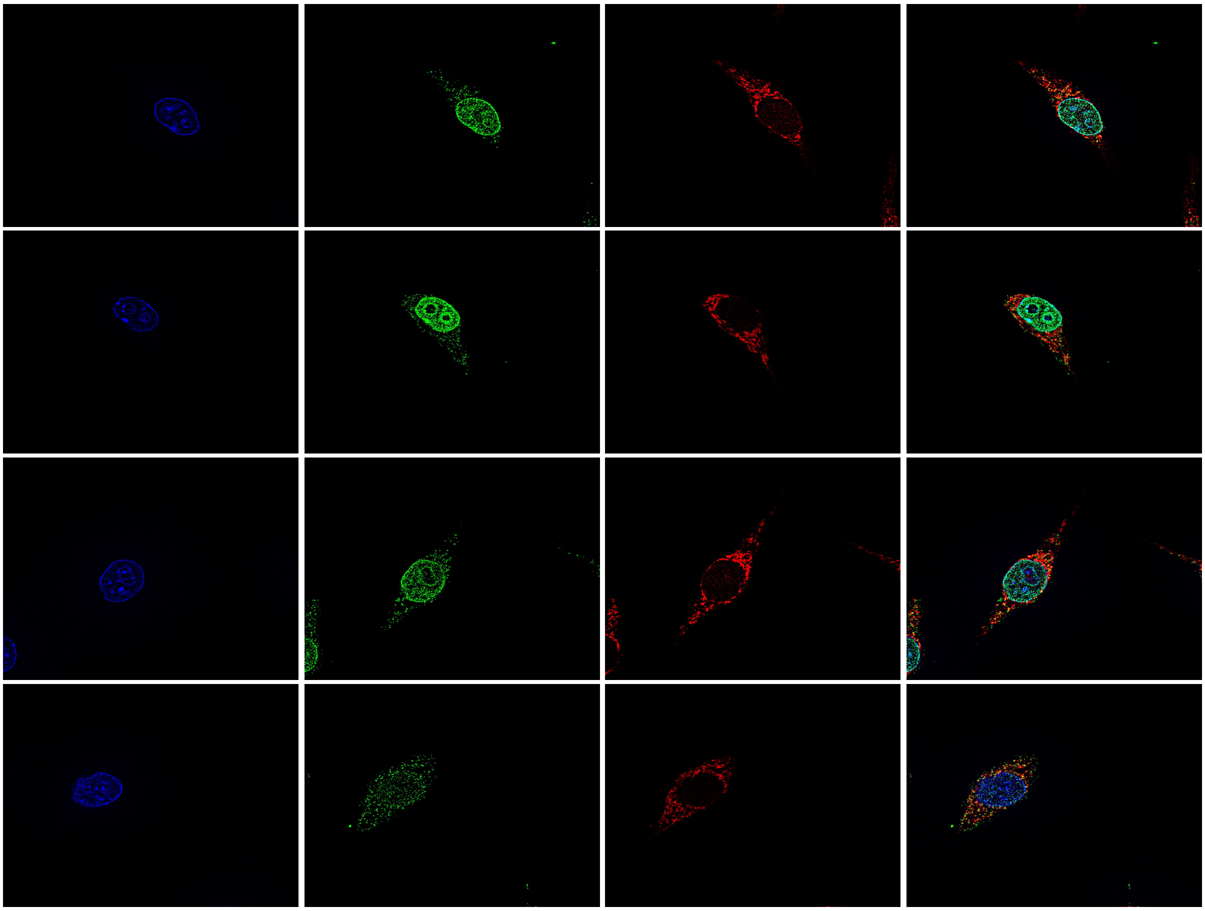

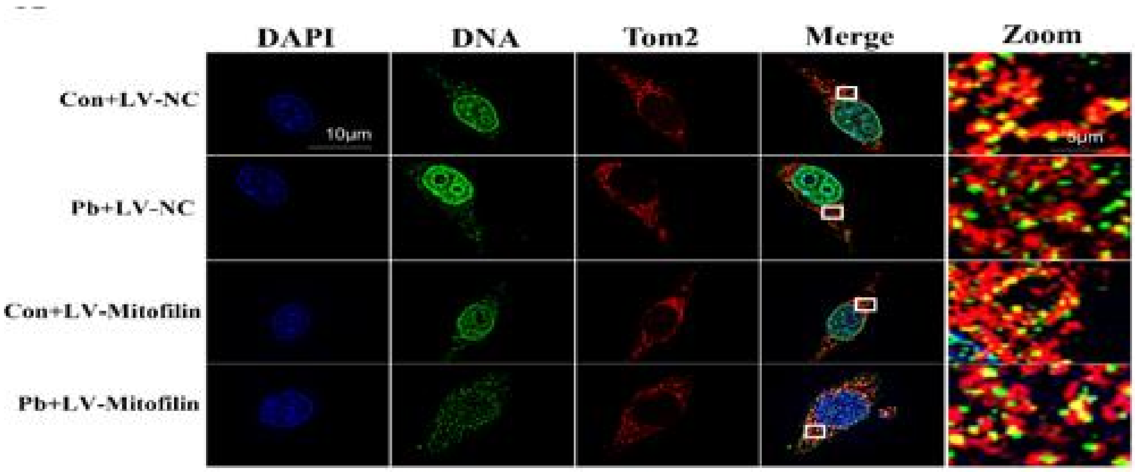

Golgi staining

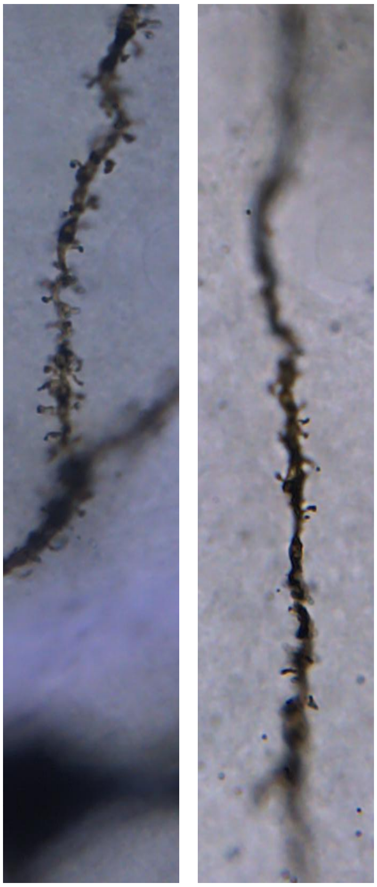

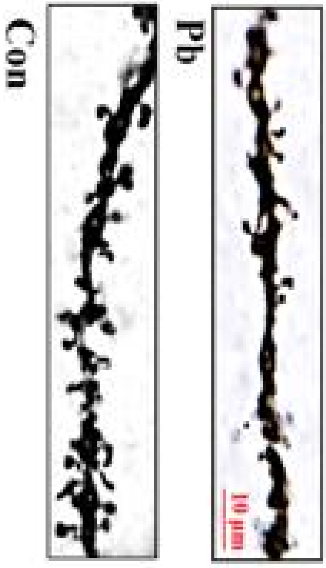

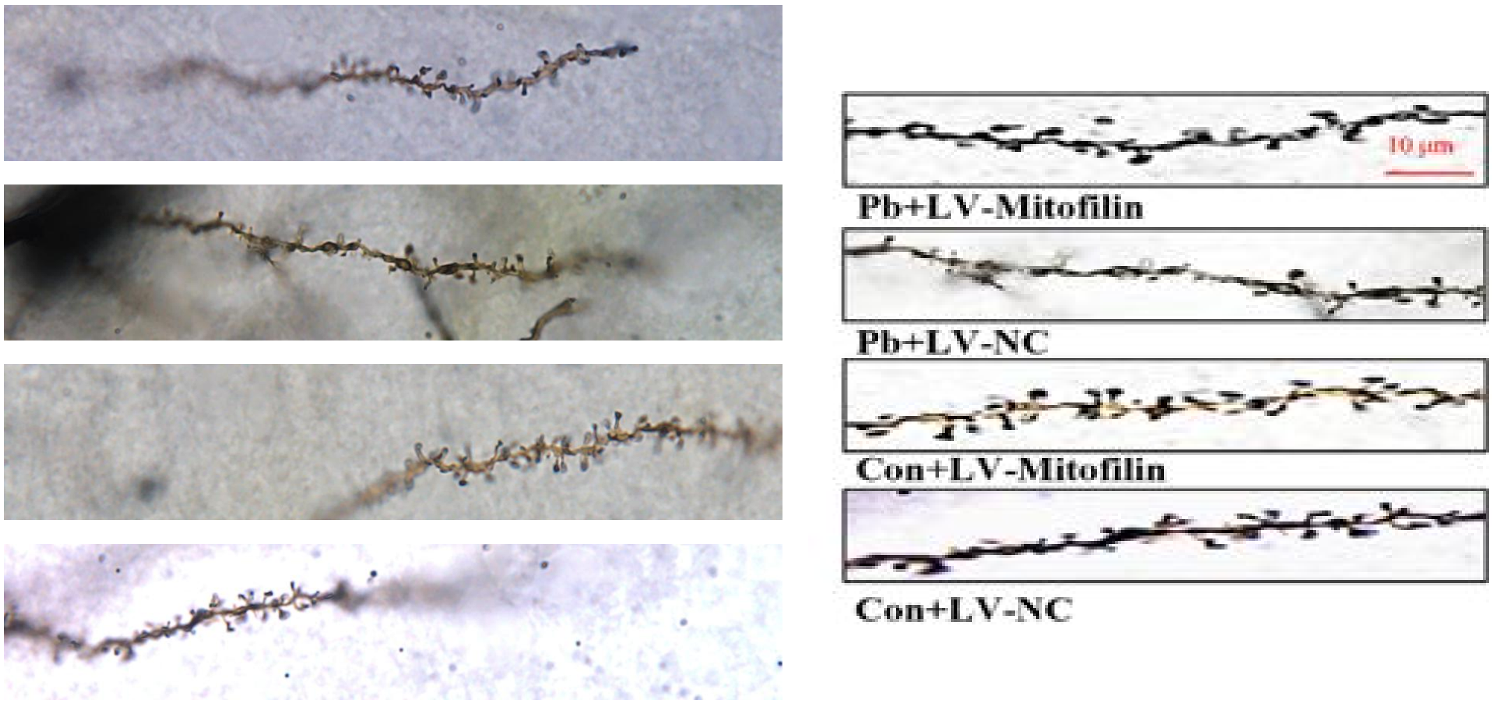
10 μm
Pb+LV-Mitofilin
Pb+LV-NC
Con+LV-Mitofilin
Con+LV-NC
